# Establishment of a human embryonic stem cell line with homozygous *TP53* R248W mutant by TALEN mediated gene editing

**DOI:** 10.1016/j.scr.2018.04.013

**Published:** 2018-04-27

**Authors:** An Xu, Ruoji Zhou, Jian Tu, Zijun Huo, Dandan Zhu, Donghui Wang, Julian A. Gingold, Helen Mata, Pulivarthi H. Rao, Mo Liu, Alaa M.T. Mohamed, Celine Shuet Lin Kong, Brittany E. Jewell, Weiya Xia, Ruiying Zhao, Mien-Chie Hung, Dung-Fang Lee

**Affiliations:** a Department of Integrative Biology and Pharmacology, McGovern Medical School, The University of Texas Health Science Center at Houston, Houston, TX 77030, USA; b The University of Texas, MD Anderson Cancer Center UTHealth Graduate School of Biomedical Sciences, Houston, TX 77030, USA; c Department of Musculoskeletal Oncology, The First Affiliated Hospital of Sun Yat-Sen University, Guangzhou 510080, PR China; d Department of Endocrinology, The First Affiliated Hospital of Sun Yat-Sen University, Guangzhou 510080, PR China; e Department of Pathophysiology, Zhongshan School of Medicine, Sun Yat-Sen University, Guangzhou 510080, PR China; f Women’s Health Institute, Cleveland Clinic Foundation, Cleveland, OH 44195, USA; g Department of Pediatrics, Baylor College of Medicine, Texas Children’s Cancer and Hematology Centers, Houston, TX 77030, USA; h Department of Molecular and Cellular Oncology, The University of Texas M.D. Anderson Cancer Center, Houston, TX 77030, USA; i Graduate Institute of Biomedical Sciences and Center for Molecular Medicine, China Medical University, Taichung 404, Taiwan; j Center for Stem Cell and Regenerative Medicine, The Brown Foundation Institute of Molecular Medicine for the Prevention of Human Diseases, The University of Texas Health Science Center at Houston, Houston, TX 77030, USA; k Center for Precision Health, School of Biomedical Informatics and School of Public Health, The University of Texas Health Science Center at Houston, Houston, TX 77030, USA

## Abstract

Genetic mutations in *TP53* contribute to multiple human cancers. Here we report the generation of a H1-p53(R248W/R248W) human embryonic stem cell line harboring a homozygous *TP53* R248W mutation created by TALEN-mediated precise gene editing. The H1-p53(R248W/R248W) cell line maintains a normal karyotype, robust pluripotency gene expression, and the potential to differentiate to the three germ layers.

**Table:** Resource table.

Unique stem cell line identifier	WAe001-A-17
Alternative name(s) of stem cell line	H1-p53(R248W/R248W) and 1P6
Institution	The University of Texas Health Science Center at Houston, Houston, Texas, USA
Contact information of distributor	Dung-Fang Lee dung-fang.lee@uth.tmc.edu
Type of cell line	Human embryonic stem cell line
Origin	Human
Additional origin info	Sex: Male
Cell Source	H1 hESCs
Clonality	Clonal
Method of reprogramming	N/A
Genetic Modification	Yes
Type of Modification	Homozygous R248W mutation of *TP53* on exon 7
Associated disease	Li-Fraumeni syndrome; cancers
Gene/locus	17p13.1; *TP53* exon 7
Method of modification	Transcription activator-like effector nucleases (TALEN)
Name of transgene or resistance	N/A
Inducible/constitutive system	N/A
Date archived/stock date	2017/12
Cell line repository/bank	Human Pluripotent Stem Cell Registry (https://hpscreg.eu/user/cellline/edit/WAe001-A-17)
Ethical approval	Cell lines were used according to institutional guidelines. UTHealth approval number: SCRO-16-01

## Resource utility

Growing evidence underscores the important contributions of p53 mutations in driving tumor progression. The H1-p53(R248W/R248W) line provides an ideal model for studying the functions of the p53(R248W) mutation in tumor initiation and progression in a human cell model, which opens up opportunities for development of new cancer therapeutic strategies.

## Resource detail

*TP53* is the most mutated gene in human cancers and leads to tumor initiation and progression in multiple cell types (Zhou et al., 2017). Previously, we established a Li-Fraumeni syndrome (LFS) iPSC disease model to investigate LFS-associated osteosarcoma (Lee et al., 2015), demonstrating the potential of a pluripotent stem cell (PSC) disease model to study mutant p53 (mutp53)-associated malignancies. Generation of disease-related genetic traits in PSCs provides an attractive approach to elucidate gene function in disease development (Gingold et al., 2016). We recently generated a H1-p53(R282W/R282W) line as a laboratory resource to facilitate use of the PSC disease model in studying p53(R282W)-associated malignancies (Zhou et al., 2018).

**Fig. 1:**
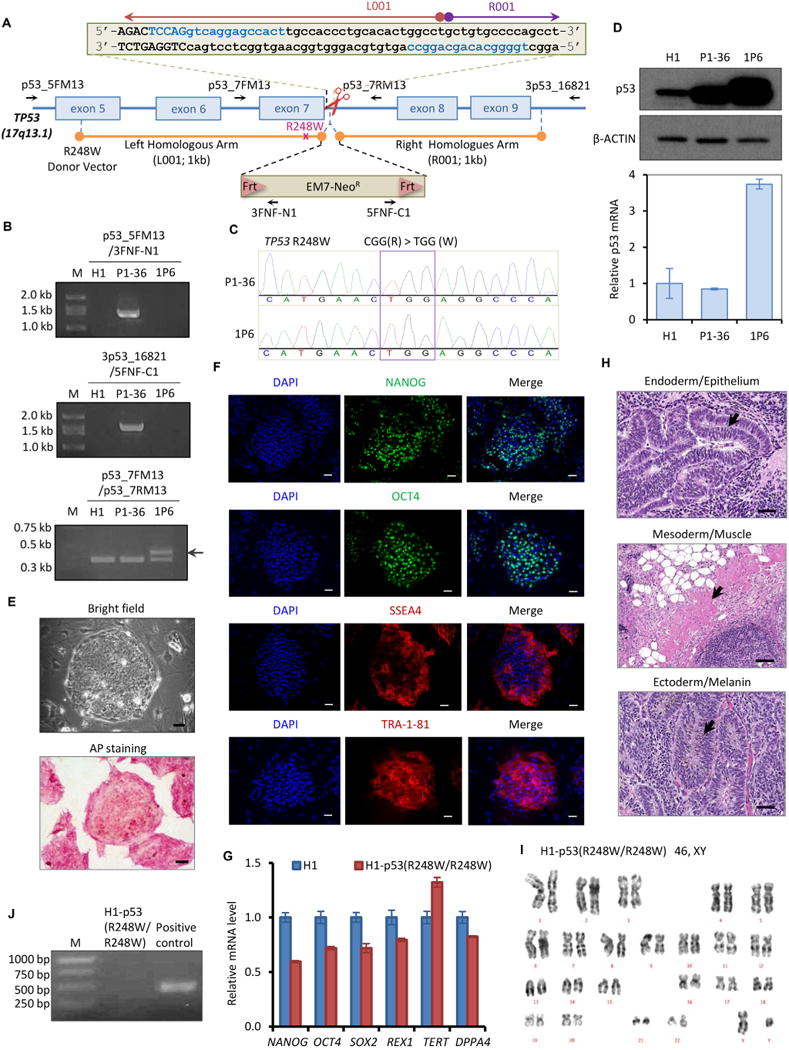
Generation and characterization of the homozygous p53 R248W mutant human embryonic stem cell line H1–p53(R248W/R248W). (A) Schematic overview of the strategy for *TP53* R248W mutation generation in the *TP53* genomic locus. TALEN target sites sequences are shown in blue. Exon sequences are in upper case, while intron sequences are in lower case. A Frt-EM7-NeoR-Frt (FNF) cassette is introduced between the left homologous arm (containing the R248W mutation) and the right homologous arm, as indicated by rods. PCR primers used for validation and sequencing are indicated with arrows. (B) Precise homologous recombination in the *TP53* genomic locus. P1–36 and 1P6 clones were confirmed by genomic PCR. Arrow indicates the footprint of Frt. (C) Sanger sequencing indicates the presence of the R248W mutation in both alleles of H1–p53(R248W/R248W) line. (D) Western blotting reveals increased protein stability of p53(R248W) in the H1–p53(R248W/R248W) line (upper panel). qRT-PCR assay indicates increased p53 mRNA expression in the H1–p53(R248W/R248W) line (lower panel). (E) Cell morphology (upper panel) and AP staining (lower panel) of the H1–p53(R248W/R248W) line. Scale bar, 50 μm. (F) Immunofluorescence staining of pluripotency factors (NANOG and OCT4) and hESC surface markers (SSEA4 and TRA-1-81) in the H1–p53(R248W/R248W) line. Scale bar, 50 μm. (G) qRT-PCR assay for the expression of endogenous pluripotency genes (*NANOG, SOX2, OCT4, DPPA4, REX1*, and *TERT*) in the H1–p53(R248W/R248W) line. (H) *In vitro* teratoma assay of the H1–p53(R248W/R248W) line. Scale bar, 50 μm. (I) Karyotype analysis of the H1–p53(R248W/R248W) line. (J) Mycoplasma PCR detection of the H1–p53(R248W/R248W) line.

To enhance our understanding of the landscape of mutp53-associated malignancies, we generated a H1 hESC line harboring a homozygous *TP53* R248W mutation. The strategy of TALEN-mediated precise gene editing is illustrated in Fig. 1A. The targeting plasmids contain (1) a pair of TALEN plasmids targeting exon7 and intron 7 of *TP53*, respectively; and (2) a pFNF donor vector carrying a Frt-EM7-Neo^R^-Frt (FNF) selection cassette flanked by 1 kb left and right homologous arms of the *TP53* genomic region. H1 hESCs were transduced with these plasmids and selected by G418. G418-resistant clones were confirmed by genomic PCR using two pairs of primers (p53_5FM13 and 3FNF-N1 for left arm, 5FNF-C1 and 3p53_16821 for right arm, Table 2) (Fig. 1B). Clone P1–36 demonstrated accurate insertion of the FNF cassette with a *TP53* R248W mutation into the *TP53* genome targeting site. Unexpectedly, Sanger sequencing of endogenous *TP53* exon 7 (using the pair of primers p53_7FM13 and p53_7RM13) in the P1–36 line revealed the presence of a homozygous *TP53* R248W mutation in which the mutation was also detected in the un-inserted allele (Fig. 1C, upper panel). The unique DNA sequence chromatogram of TGG in P1–36 ruled out the possibility of a mixed population in P1–36 (Fig. 1C, upper panel). This additional *TP53* R248W mutation was probably created spontaneously during mutant donor vector-mediated homologous recombination.

The FNF cassette in the P1–36 clone was subsequently removed by transfecting the line with Flp recombinase plasmid. The FNF removal clone 1P6 was identified by loss of genomic PCR products (Fig. 1B, upper and middle panels). The larger PCR band of the *TP53* exon 7 region of the FNF-removed clone 1P6 demonstrated the footprint of Frt, which was all that remained after excision from the FNF cassette-inserted allele (Fig. 1B, arrow in lower panel). Sanger sequencing of endogenous *TP53* exon 7 of the 1P6 clone further confirmed the presence of the *TP53* R248W mutation in both alleles (Fig. 1C, lower panel), consistent with the findings from the parental P1–36 clone. Im-munoblotting revealed higher p53 protein levels in both P1–36 and 1P6 clones than those in parental H1 line (Fig. 1D, higher panel), supporting the increased protein stability of mutp53. Although both P1–36 and 1P6 clones demonstrated homozygous *TP53* R248W mutation, higher p53 mRNA levels were measured in the 1P6 clone, explaining the higher p53 protein levels in the 1P6 clone compared to the P1–36 clone (Fig. 1D, lower panel). We rename the 1P6 line to H1-p53(R248W/R248W).

The H1-p53(R248W/R248W) line maintained a classical dome-shaped hESC morphology and exhibited positive alkaline phosphatase (AP) activity (Fig. 1E, scale bar 50 μm). Immunofluorescent staining of the H1-p53(R248W/R248W) line demonstrated high expression of hESC pluripotency factors and hESC surface markers (Fig. 1F, scale bar 50 μm). Although quantitative real-time PCR (qRT-PCR) showed lower mRNA levels of pluripotency genes in the H1-p53(R248W/R248W) line compared with the parental H1 line (Fig. 1G), these cells functionally perform as PSCs, as demonstrated by their proliferation in hESC medium as well as *in vivo* three germ-line differentiation capacity (Fig. 1H, scale bar 50 μm). Karyotype analysis confirmed the normal karyotype of the H1-p53(R248W/R248W) line (Fig. 1I). Furthermore, PCR-based mycoplasma detection assay demonstrated that the H1-p53(R248W/R248W) line is mycoplasma-free (Fig. 1 J). The short tandem repeat (STR) profile of H1-p53(R248W/R248W) line was identical to that of its parental H1 line (data available with authors). The characterization of the H1-p53(R248W/R248W) line was summarized in Table 1.

In summary, the H1-p53(R248W/R248W) line is karyotypically normal and retains pluripotency. This line has great potential to offer insight into the role of p53(R248W) in embryogenesis and tumorigenesis.

## Materials and methods

### Cell culture

Cell culture of hESC H1 and H1-derived clones were performed as described (Zhou et al., 2018).

### Construct of TALEN plasmids and TP53(R248W) donor vector

The TALEN guides targeting intron 7 of *TP53* were designed by ZiFiT Targeter Version 4.2 (http://zifit.partners.org/). Each TAL effector binding domain targeting upstream (5′-TCCAGGTCAGGAGCC ACT-3′) and downstream (5′-TGGGGCACAGCAGGCC-3′) were assembled into JDS vectors according to the protocol developed by the Joung Lab (Sander et al., 2011).

The left and right homologous arms were amplified from hESC H1 gDNA isolated using PureLink Genomic DNA Mini Kit (Thermo Fisher) (Table 2). PCR products of both arms were separated on 0.8% agarose gel, extracted by QIAquick gel extraction kit (Qiagen), ligated into pGEM-Teasy vector (Promega), and confirmed by Sanger sequencing. Site-directed mutagenesis was performed to generate the R248W mutation on the left homologous arm. Both homologous arms were digested by designed restriction enzymes (*Eco*RI used for the right homologous arm and *Bam*HI and *Not*I used for the left homologous arm) and ligated into the pFNF (Frt-EM7-Neo^R^-Frt) vector (Addgene plasmid #22687) to generate the *TP53* R248W donor vector.

### Generation of H1-p53(R248W/R248W) line by TALEN-mediated genome editing

One day before electroporation, irradiated CF1 MEFs (Thermo Fisher) were seeded on 0.1% gelatin pre-coated dishes using MEF culture medium (DMEM supplemented with 10% of FBS (GenDEPOT) and 1% Penicillin/Streptomycin) at a density of 6.7 × 10^5^ cells per 100 mm dish.

To generate the H1-p53(R248W/R248W) cell line, 1 × 10^7^ H1 hESCs were re-suspended in 0.6 ml Embryo Max Electroporation Buffer (Millipore) mixed with 50 μg of *TP53* R248W donor vector and 5 μg of each TALEN plasmids, and electroporated at 300 V/500 uF in the Bio-Rad Gene Pulser Xcell System. After electroporation, cells were immediately seeded on 100 mm MEF plates in hESC medium (DMEM/F12 (Corning) supplemented with 20% KnockOut Serum replacement (Life Technologies), 1% Gibco GlutaMax (Life Technologies), 1% NEAA (Corning), 0.0007% β-mercaptoethanol (Sigma) and 10 ng/ml FGF2 (EMD Millipore)) supplemented with 2 μM ROCK inhibitor Thiazovivin. After 2 days, cells were selected with 50 μg/ml G418 for 2–3 weeks and medium was changed every 2 to 3 days until colonies were big enough for picking. Individual clones were picked and expanded for further confirmation by genomic PCR as described above.

### Standard PCR

Genomic DNA was isolated by Easy-DNA gDNA purification kit (Invitrogen) according to manufacturer’s instructions. OneTaq Quick-Load 2× Master Mix (New England Biolabs) was used for standard PCR according to manufacturer’s instructions. Reaction was performed as following protocol: 94 °C for 1 min; 35 cycles of reaction: 94 °C for 30 s, 56 °C for 45 s and 68 °C for 1 min; and 68 °C for 5 min on a Biometra TRIO Thermal Cyclers (Analytik Jena). The product sizes of PCR are 1370 bp using p53_5FM13 and 3FNF-N1 primers, 368 bp and 432 bp (with Frt footprint) using p53_7FM13 and p53_7RM13 primers, and 1606 bp using 3p53_16821 and 5FNF-C1 primers.

### Western blotting analysis and immunofluorescent staining

Immunoblotting and immunofluorescent staining were performed as described (Zhou et al., 2018).

### qRT-PCR

Total mRNA from the H1-p53(R248W/R248W) cells was extracted using TRIzol (Invitrogen). Reverse Transcription cDNA synthesis reactions were performed by iScript cDNA synthesis kit (Bio-Rad). Quantitative PCR reactions were performed using SYBR Green PCR Master Mix (Bio-Rad) on a CFX96 machine (Bio-Rad). The reaction was performed as following parameters: 50 °C for 10 min, 95 °C for 5 min, 40 cycle of 95 °C for 10 s and 60 °C for 30 s, and 95 °C for 10 min. Samples were analyzed in triplicate and normalized to GAPDH expression.

### Karyotype analysis

The G-banding karyotype was carried out in the Department of Pediatrics, Baylor College of Medicine, Texas Children’s Cancer and Hematology Centers. Twenty metaphase chromosome spreads were counted and G band resolution was 400. The H1-p53(R248W/R248W) line were karyotyped at passage 5 after the line was generated.

### In vivo teratoma formation assay

Teratoma formation assay was performed as described (Zhou et al., 2018).

### Mycoplasma detection

Mycoplasma detection was performed using PCR Mycoplasma Detection Kit (Applied Biological Materials Inc).

### STR analysis

STR analysis was performed by the Characterized Cell Line Core Facility at the University of Texas M.D. Anderson Cancer Center. The number of STRs at 14 loci (AMEL, CSF1PO, D13S317, D16S539, D18S51, D21S11, D3S1358, D5S818, D7S820, D8S1179, FGA, TH01, TPOX and vWA) was assessed for H1-p53(R248W/R248W) cells. This STR profile was compared with the STR profile of parental H1 cells.

## Figures and Tables

**Table 1:** Characterization and validation.

Classification	Test	Result	Data
Morphology	Photography	hESC morphology	Fig. 1 panel E
Phenotype	Immunocytochemistry	NANOG, OCT4, SSEA4, TRA-1-81 and AP-positive	Fig. 1 panel E, F
	RT-qPCR	Lower levels of expression of *NANOG*, *OCT4*, *SOX2*, *DPPA4* and *REX1* compared with H1	Fig. 1 panel G
Genotype	Karyotype (G-banding) and resolution	46 XY Resolution: 400	Fig. 1 panel I
Identity	Microsatellite PCR (mPCR) OR	N/A	N/A
	STR analysis	14 sites tested, STR profile matches human embryonic cell line H1	Data available with authors
Mutation analysis (IF APPLICABLE)	Sequencing	Homozygous R248W mutation of *TP53*.	Fig. 1 panel C
	Southern Blot OR WGS	N/A	N/A
Microbiology and virology	Mycoplasma	Mycoplasma test shows negative.	Fig. 1 panel J
Differentiation potential	*In vivo* teratoma	Teratoma comprises tissues of ectoderm, mesoderm, and endoderm.	Fig. 1 panel H
Donor screening (OPTIONAL)	HIV 1 + 2 Hepatitis B, Hepatitis C	N/A	N/A
Genotype additional info (OPTIONAL)	Blood group genotyping	N/A	N/A
	HLA tissue typing	N/A	N/A

**Table 2:** Reagents details.

Antibodies used for immunocytochemistry			
	Antibody	Dilution	Company Cat # and RRID
Pluripotency Markers	Goat anti-NANOG	1:500	R and D Systems Cat# AF1997 RRID:AB_355097
Pluripotency Markers	Rabbit anti-OCT4	1:300	Santa Cruz Biotechnology Cat# sc-9081 RRID:AB_2167703
Pluripotency Markers	Mouse anti-SSEA4 PE-conjugated	1:600	R and D Systems FA1435P-025
Pluripotency Markers	Mouse anti-TRA-1-85 Alexa Fluor 555-conjugated	1:600	R and D Systems Cat# FAB3195A RRID:AB_663789
p53(Western Blotting)	Mouse anti-p53 (DO-1)	1:1000	Santa Cruz Biotechnology Cat# sc-126
B-ACTIN (Western Blotting)	Mouse anti-β-ACTIN	1:10000	Proteintech Cat#66009–1-Ig
Secondary antibodies	Goat anti-rabbit IgG (Alexa Fluor 488 conjugate)	1:500	Jackson ImmunoResearch Labs Cat# 111–545-144 RRID:AB_2338052
Secondary antibodies	Donkey Anti-Goat IgG (Alexa Fluor488 conjugate)	1:500	Jackson ImmunoResearch Labs Cat# 705–545-003 RRID:AB_2340428

Primers			
	Target	Forward/Reverse primer (5′–3′)	

Pluripotency Markers (qPCR)	*OCT4*	AACCTGGAGTTTGTGCCAGGGTTT/TGAACTTCACCTTCCCTCCAACCA	
Pluripotency Markers (qPCR)	*SOX2*	AGAAGAGGAGAGAGAAAGAAAGGGAGAGA/GAGAGAGGCAAACTGGAATCAGGATCAAA	
Pluripotency Markers (qPCR)	*NANOG*	TTTGTGGGCCTGAAGAAAACT/AGGGCTGTCCTGAATAAGCAG	
Pluripotency Markers (qPCR)	*DPPA4*	GACCTCCACAGAGAAGTCGAG/TGCCTTTTTCTTAGGGCAGAG	
Pluripotency Markers (qPCR)	*REX1*	GCCTTATGTGATGGCTATGTGT/ACCCCTTATGACGCATTCTATGT	
Pluripotency Markers (qPCR)	*TERT*	TGAAAGCCAAGAACGCAGGGATG/TGTCGAGTCAGCTTGAGCAGGAATG	
p53 (qPCR)	*TP53*	CAGCACATGACGGAGGTTGT/CCAGACCATCGCTATCTGAGC	
Housekeeping Genes (qPCR)	*GAPDH*	CCACTCCTCCACCTTTGAC/ACCCTGTTGCTGTAGCCA	
Targeted mutation analysis/sequencing	p53_5FM13	TGTAAAACGACGGCCAGTCTAGCTCGCTAGTGGGTTGC	
Targeted mutation analysis/sequencing	3FNF-N1	TCCAGACTGCCTTGGGAAA	
Targeted mutation analysis/sequencing	5FNF-C1	GGGGAGGATTGGGAAGACAA	
Targeted mutation analysis/sequencing	3p53_16821	CAGGAAACAGCTATGACCGCCCAGGAGGGTATAATGAGCTA	
Targeted mutation analysis/sequencing	p53_7FM13	TGTAAAACGACGGCCAGTGCCTCCCCTGCTTGCCACAG	
Targeted mutation analysis/sequencing	p53_7RM13	CAGGAAACAGCTATGACCGGGAGCAGTAAGGAGATTCC	
Targeted mutation analysis/sequencing	5p53-L001-1 kb/EcoRI for left homologous arm	GAATTCCGCGTCCGCGCCATGGCCATCTACAAGCAGTCACAG	
Targeted mutation analysis/sequencing	3p53-L001-1 kb/EcoRI for left homologous arm	GAATTCAGGCCAGTGTGCAGGGTGGCAAGTGGCTCCTGACCT	
Targeted mutation analysis/sequencing	5p53-R001-1 kb/BamHI for right homologous arm	GGATCCGCTGTGCCCCAGCCTCTGCTTGCCTCTGACCCCTGG	
Targeted mutation analysis/sequencing	3p53-R001-1 kb/NotI for right homologous arm	GCGGCCGCCAGGCTAGGCTAAGCTATGATGTTCCTTAGATTAGG	
Targeted mutation analysis/sequencing	5p53-R248W for R248W mutagenesis	TGCATGGGCGGCATGAACTGGAGGCCCATCCTCACCATC	
Targeted mutation analysis/sequencing	3p53-R248W for R248W mutagenesis	GATGGTGAGGATGGGCCTCCAGTTCATGCCGCCCATGCA	
